# Perinephric myxoid pseudotumor of fat: the first case report in China with an updated literature review

**DOI:** 10.3389/fonc.2025.1543068

**Published:** 2025-04-16

**Authors:** Yu-Hang Yang, Yue-Ming Zhang, An-Hao Liu, Guang-Cheng Luo, Zheng-Jin Liu, Jian-Wei Xie

**Affiliations:** ^1^ Zhongshan Hospital of Xiamen University, School of Medicine, Xiamen University, Xiamen, China; ^2^ Department of Urology, Zhongshan Hospital of Xiamen University, School of Medicine, Xiamen University, Xiamen, China; ^3^ Department of Pathology, Zhongshan Hospital of Xiamen University, School of Medicine, Xiamen University, Xiamen, China

**Keywords:** perinephric myxoid pseudotumor of fat, renal disease, MDM2, pathology, diagnosis

## Abstract

The perinephric myxoid pseudotumor of fat (PMPF) is an uncommon benign neoplasm characterized by a favorable prognosis. To date, 21 cases of PMPFs have been reported worldwide, and their detailed characteristics have not been fully elucidated. We report the 22nd case of PMPF, which is the first case in China and happened in a 54-year-old male with a 5.6 cm mass located in the lower inner aspect of the right kidney. The patient underwent a minimally invasive robot-assisted laparoscopic resection of the renal mass. Postoperative histopathological, immunohistochemical, and genetic testing results confirmed the diagnosis of PMPF. No evidence of recurrence was found during a follow-up period of 6 months postoperatively based on clinical and imaging data. This is a report of an unusual case of PMPF with an up-to-date review. According to the latest systematic review and clinical confirmation, PMPF is strongly associated with chronic kidney disease, which is further confirmed by our case. For PMPF, combining imaging methods and immunohistochemical staining is suggested, which may prove beneficial in clinical practice.

## 1 Introduction

The perinephric myxoid pseudotumor of fat (PMPF) is an uncommon retroperitoneal mass composed of myxoid lipomatous tissue, initially described by Tanas et al. as a fibroblastic or myofibroblastic proliferation in the perinephric adipose tissue adjacent to renal cell carcinoma in 2009 (1). Subsequently, Dashti et al. identified PMPF in a pathological series of 11 perirenal masses and observed its occurrence primarily in patients with non-neoplastic kidney diseases (2). To date, a total of 21 cases of PMPF have been documented globally (2–8).

Although the PMPF is generally characterized by a favorable prognosis, its diagnosis may confuse the pathologists as its detailed characteristics have not been fully elucidated. This article presents the 22nd case of PMPF globally, which is the first case of PMPF in China, and provides a more comprehensive review of previous case reports, aiming to deepen the understanding of this disease.

## 2 Case report

**Figure 1:**
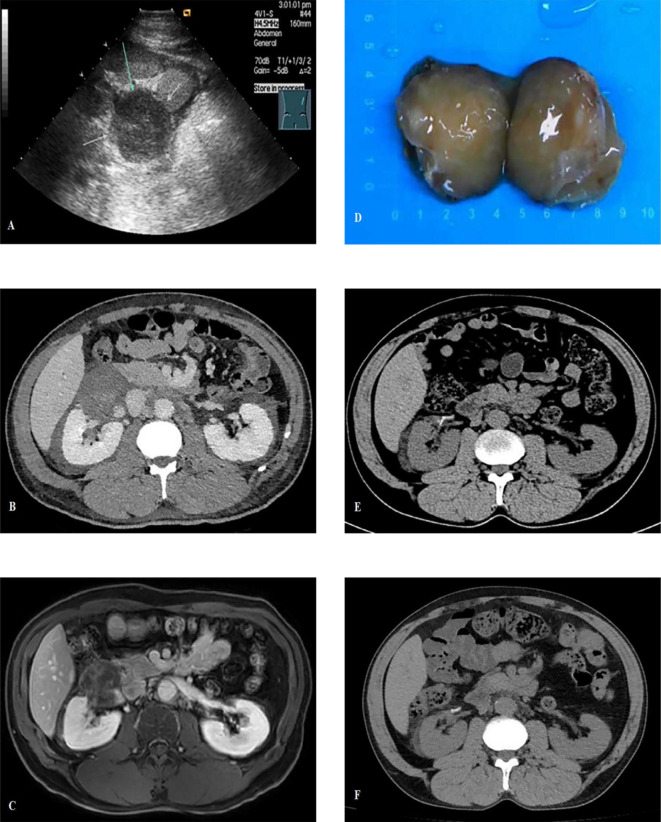
**(A)** Urological ultrasound imaging revealed an irregular hypoechoic mass measuring approximately 5.6cm with no internal blood flow signals; **(B)** The contrast-enhanced computed tomography (CT) demonstrated renal artery tortuosity and a distinct demarcation between the mass and renal parenchyma; **(C)** The contrast-enhanced MRI revealed delayed phase nodular enhancement with long T2 signal intensity; **(D)** The tumor gross specimen presents as a well-defined 6cm brownish-yellow lobulated mass, exhibiting no indications of hemorrhage or necrosis; **(E)** The postoperative CT scan showed no abnormality; **(F)** The CT scan at 6 months post-surgery shows no recurrence.

In 2019, urological ultrasound imaging revealed an irregular hypoechoic mass measuring approximately 5.6 cm in the lower inner aspect of the right kidney in a 54-year-old male patient with a medical history of stage IV lupus nephritis and stage III chronic kidney disease (CKD). The mass exhibited peripheral vascularity, while no internal blood flow signals were detected in ultrasound. (**Figure 1A**). The contrast-enhanced computed tomography findings did not reveal any significant tumor enhancement; however, they demonstrated renal artery tortuosity and a distinct demarcation between the mass and renal parenchyma (**Figure 1B**), indicating the presence of a cystic lesion adjacent to the right renal pelvis classified as Bosniak IIF type. Laboratory tests revealed normal ranges of carcinoembryonic antigen (CEA), cancer antigen 125, cancer antigen 15-3, cancer antigen 19-9, prostate-specific antigen (PSA), and free PSA/total PSA ratio. Subsequently, our team conducted longitudinal monitoring of the right renal cystic lesion. The contrast-enhanced magnetic resonance imaging of the kidney in 2023 revealed delayed phase nodular enhancement with long T2 signal intensity (**Figure 1C**), leading to a diagnosis of Bosniak IV type complex cyst in the right kidney.

Based on the above results, our team initially considered it as a complex renal cyst but could not rule out the possibility of malignancy. After being informed, the patient and their family requested surgical treatment. The surgery was conducted utilizing a robot-assisted laparoscopic technique. The tumor presented as a well-defined 6 cm brownish-yellow lobulated mass, exhibiting no indications of hemorrhage or necrosis (**Figure 1D**).

**Figure 2:**
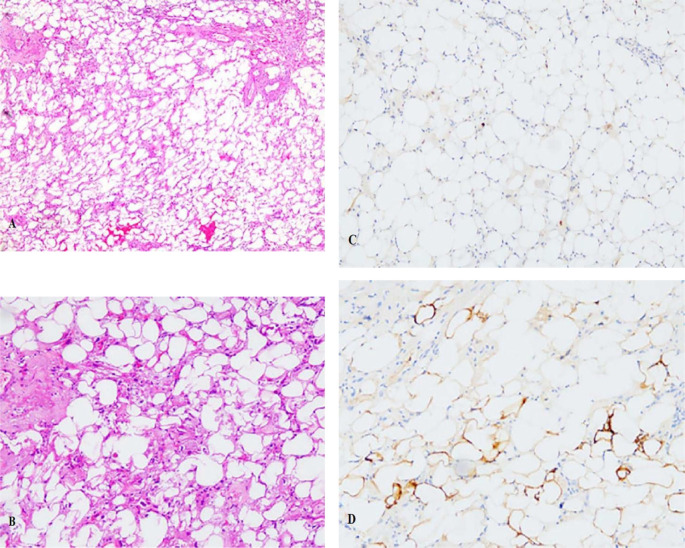
**(A)** The HE staining revealed the presence of cellular atypia in certain cells, accompanied by adipocytic differentiation and infiltration of lymphocytes and plasma cells (×5); **(B)**The HE staining for cells (×10); **(C)** Immunohistochemistry staining revealed a positive result for MDM2 in certain cellular populations; **(D)** Immunohistochemistry staining revealed a negative result for S100.

Tissue samples were submitted to the pathology department for subsequent histological examination. The microscopic analysis revealed the presence of cellular atypia in certain cells, accompanied by adipocytic differentiation and infiltration of lymphocytes and plasma cells (**Figures 2A, B**). The formation of a central hair follicle region was also observed. Immunohistochemistry staining revealed a positive result for MDM2 (**Figure 2C**) in certain cellular populations and a negative result for S-100 (**Figure 2D**). Preliminary consideration was highly differentiated liposarcoma. After communicating with the patient and his family members, the tumor tissue was also sent to Xiamen Eda Medical Laboratory for genetic testing. The test results revealed no amplification of the MDM2 gene, the CDK4 gene, or breakage in the DDIT3 gene. Obviously inconsistent with the previous diagnosis, the tumor tissue was sent to the superior oncology specialist hospital for pathology consultation, which showed that the MDM2 and CDK4 genes were not amplified. Combining genetic testing and immunohistochemistry in a superior hospital, the obtained results enabled the classification as PMPF.

**Figure 3:**
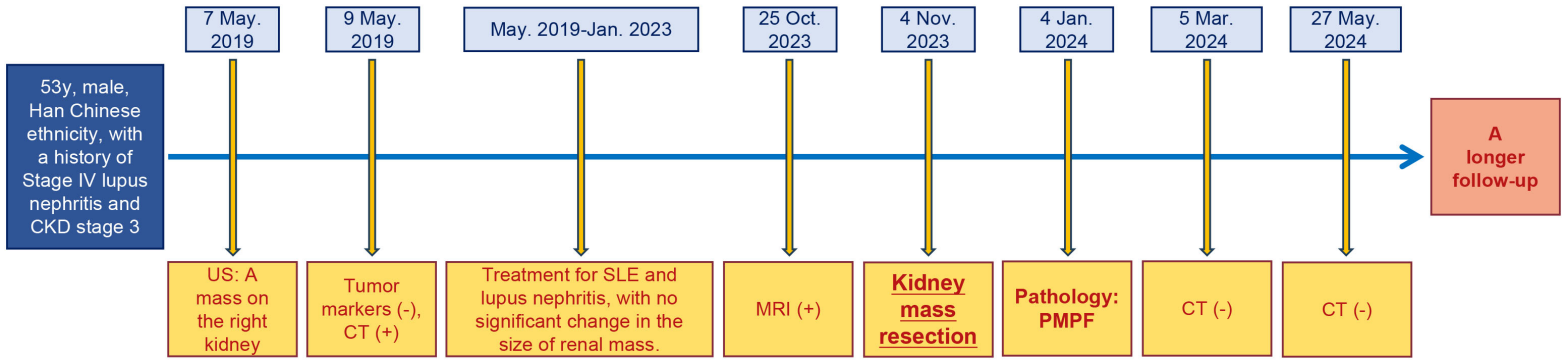
The timeline of this case report.

Clinical and imaging follow-up (**Figures 1E, F**) at 6 months after surgery revealed a good clinical outcome for the patient, and a longer-term follow-up was also required. Additionally, the timeline of this case report is shown in **Figure 3**.

## 3 Discussion

In clinical practice, PMPF is a rare disease presenting a benign tumor biomorphology and usually without obvious clinical manifestations. It is prevalent in middle-aged and older male individuals with underlying chronic renal-related conditions such as chronic renal insufficiency, lupus nephritis, pyelonephritis and diabetes. Perinephric myxoid lipofibroma comprises mature adipose tissue, a myxoid matrix, spindle-shaped to star-shaped stromal cells, and varying degrees of mixed inflammatory cell infiltration. The mixed inflammatory cells mainly consist of lymphocytes and small clusters of plasma cells (2–8).

Due to the presence of various benign and malignant lesions in renal parenchymal soft tissue, the differential diagnosis of PMPF includes conditions such as renal lipoma, retroperitoneal fibromatosis, renal perivascular leiomyomatous lipoma, medullary lipoma, IgG4-related renal disease, and liposarcoma (9, 10). Renal lipomas are characterized by adipose tissue proliferation in the renal sinus, hilum, and perinephric space without the presence of moderately atypical mesenchymal cells observed in PMPF. Retroperitoneal fibromatosis is composed of spindle-shaped fibroblasts, myoblasts, and collagen bundles arranged in long ribbon-like structures with some areas exhibiting a wavy appearance. The diagnosis of fibromatosis can be supported by positive nuclear staining with β-catenin or molecular genetic studies indicating CTNNB1 mutations (11, 12).

Renal vascular smooth muscle fatty tumor is a neoplasm composed of abnormal proliferation of vascular, smooth muscle, and adipose tissues with varying proportions of each component, often predominantly consisting of adipose tissue. The tumor exhibits expansive growth and lacks invasiveness. HMB45 and Melan-A immunohistochemistry can be utilized to differentiate it from vascular leiomyofibroma. Medullary fatty tumor consists of mature adipose tissue and hematopoietic tissue that can be easily distinguished from perirenal myxoid lipofibroma by histopathology. IgG4-related disease is a fibroinflammatory condition characterized by tumor-like lesions, dense infiltration of IgG4+ plasma cells, sheet-like fibrosis, proliferation of ectopic germinal centers, and elevated serum levels of IgG4 (13). Among the affected organs in IgG4-related disease (IgG4-RD), the kidney is commonly involved (IgG4-related kidney disease or IgG4-RKD) (14–16). PMPF can be distinguished from IgG4-RKD based on serum levels of IgG4 >135 mg/dL, presence of >10 IgG4+ plasma cells per high-power field, and an IgG4+/IgG+ plasma cell ratio exceeding 40% (17). In our case study, there was no significant increase in the number of IgG4+ plasma cells observed; therefore, the likelihood of having an underlying diagnosis of IgG-Related Disease is low.

For the differential diagnosis of malignant tumors, liposarcoma should be considered as the most crucial factor. Myxoid liposarcoma is commonly found in the extremities and less frequently in the retroperitoneum (18). It consists of various stages of adipocyte precursor cells, branching capillaries, and a myxoid matrix. The presence of DDIT3 gene alteration can aid in diagnosing myxoid/round cell liposarcoma (with over 95% of cases showing CHOP/FUS fusion) (19). Fluorescence *in situ* hybridization for MDM2 amplification can be used to differentiate atypical/highly differentiated liposarcoma and dedifferentiated liposarcoma from PMPF (20), while CDK4 gene amplification helps to differentiate atypical/highly differentiated liposarcoma from PMPF (21). It is also crucial not to overlook the occasional misdiagnosis caused by isolated cell clusters showing a positive reaction to MDM2 in pathological immunohistochemistry. In one study (22), researchers compared the performance of MDM2 immunohistochemistry (IHC) in inflammatory well-differentiated liposarcoma (WDLS/ALT) with that in fibroinflammatory diseases (such as sclerosing mesenteritis and idiopathic retroperitoneal fibrosis). The results revealed that the false positivity rates of MDM2 IHC for fibroinflammatory diseases (sclerosing mesenteritis and retroperitoneal fibrosis) were 21% (3/14) and 10% (1/10), respectively. All these false-positive cases exhibited weak positivity (1+), and the reasons for the false positives may be related to cross-reactivity or non-specific staining with non-neoplastic cells (such as fibroblasts, plasma cells, and histiocytes) within the tissue. However, no reports or cases within our study have identified MDM2/CDK4 amplification (2–8). When a solid renal space-occupying lesion is detected by color ultrasound and the diagnosis cannot be made definitively, it should be combined with urological serological tumor markers and serum IgG4 levels, and further MRI enhancement should be performed for differentiation. If the diagnosis is still difficult, an ultrasound-guided puncture biopsy can be sent for immunohistochemistry, which should focus on whether MDM2 and CDK4 are amplified and whether the DDIT3 gene is broken.

Compared to previous studies, this study provides a comprehensive summary of the clinicopathological features of each patient globally in **Table 1**. The patients included in this study had an age range of 40 to 84 years (mean age: 63 ± 13 years) and lesion sizes ranging from 2.9 to 28 cm (mean size:7.1 ± 6.8 cm). The gender ratio was 20 males to 2 females, and potential renal-related diseases encompassed lupus nephritis (2/21), chronic renal insufficiency/end-stage renal disease (12/21), diabetes (7/21), chronic pyelonephritis (1/21), malignant renal tumor (1/21) and polycystic kidney (1/21). Pathological tissue samples were obtained through biopsy (6 cases), lesion resection (3 cases), nephrectomy (12 cases), and a biopsy+nephrectomy combination procedure(1 case). None of the cases exhibited positive IgG4 plasma cells or MDM2 amplification.

**Table 1:** Clinicopathologic features of all reported cases of the perinephric myxoid pseudotumors of fat.

Author	Age (yr)	Sex	No.+Location	Size (cm)	Associated disease	Tissue obtained	IgG4+ plasma cells	MDM2 FISH	Follow-up (mo)
Dashti (2019 USA) (2)	63	M	Multi.+Bil.	4.1	DM	biopsy (n=4) mass resection (n=1) nephrectomy (n=6)	Not increased	Negative	1 mo, DOC
	69	M	Multi.+Bil.	12	DM		Not increased	Negative	24 mo, ANED*
	79	M	Multi.+Bil.	14	DM+ESRD		Not increased	Negative	4 mo, ANED
	59	M	Multi.+Bil.	16	ESRD		Not increased	Negative	48 mo, ANED
	50	M	1+LK	28	NA		Not increased	Negative	24 mo, ANED
	45	M	1+LK	6.5	ESRD		Not increased	Negative	26 mo, ANED
	43	F	1+RK	NA	CPN		Not increased	Negative	120 mo, ANED
	58	M	1+LK	2.9	No disease		Not increased	Negative	48 mo, ANED
	84	M	1+LK	15	No disease		Not increased	Negative	3 mo, ANED
	78	M	1+RK	4.5	DM		Not increased	Negative	0 mo, DOC
	75	M	1+RK	NA	UC		Not increased	Negative	3 mo, ANED
Chen (2021 USA) (3)	80	M	1+LK	7	ESRD	mass resection	Not increased	Negative	NR
Thoeni (2021 Canada) (4)	40	F	1+RK	6.5	ESRD	nephrectomy	Not increased	Negative	NR
Nguyen(2022 Japan) (5)	55	M	1+RK	NA	ESRD	nephrectomy	Not increased	Negative	NR
	59	M	1+RK	NA	ESRD	nephrectomy	Not increased	Negative	NR
Lee (2022 USA) (6)	70	M	2+RK	9.1	No disease	Biopsy	Not increased	Negative	32 mo
	60	M	1+LK	7.8	ESRD+DM	nephrectomy	Not increased	Negative	29 mo
	70	M	>10+Bil.	3.5	ESRD+DM+Lupus	Biopsy	Not increased	Negative	23 mo
	50	M	2+LK	4.5	ESRD	Biopsy+nephrectomy	Not increased	Negative	16 mo
Ortiz-Rey (2023 Spain) (7)	80	M	1+LK	NA	DM+ROC	nephroureterectomy	Not increased	Negative	NR
Michael E(2024 USA) (8)	64	M	1+LK	8.4	PK+ESRD	nephrectomy	Not increased	Negative	24mo, DOC
Current case	54	M	1+RK	5.6	LN+CKD3	mass resection	Not increased	Negative	5mo, ANED
Mean±SD	63±13			7.1±6.8					

*ANED alive with no evidence of disease. CPN Chronic pyelonephritis. CKD3 Chronic kidney disease 3. DM diabetes mellitus. DOC died of other cause. ESRD end-stage renal disease. F female. FISH fluorescence in situ hybridization. LK left kidney. LN Lupus nephritis. M male. MDM2 murine double minute 2. mo month. NA not available. NR not reported. PK polycystic kidney. RK right kidney. UC urothelial carcinoma.

At present, the etiology of PMPF is not fully clear. Based on the retrospective case data of PMPF (**Table 1**), the author believes that it is more likely to be caused by inflammatory or chronic stimulation of the kidney by non-neoplastic disease. In this study (**Table 1**), 12 out of 21 PMPF patients (57%) had chronic renal insufficiency or end-stage renal disease. This suggests that patients with CKD may be more susceptible to Perinephric Myxoid Pseudotumor of Fat (PMPF). The kidneys of CKD patients often exhibit inflammatory responses or tissue damage, which can lead to fibrosis and hyperplasia of the perinephric adipose tissue. The infiltration of inflammatory cells and the release of cytokines may stimulate the proliferation of fibroblasts and myofibroblasts in the perinephric adipose tissue, contributing to the pathological characteristics of PMPF. Additionally, the perinephric adipose tissue in CKD patients may be more susceptible to inflammation, thereby promoting the development of PMPF. Fortunately, the prognosis of PMPF seems promising, with a majority of cases demonstrating stable conditions or absence of disease following treatment.

## 4 Conclusion

This study is a unique report on PMPF and provides a comprehensive review of all previous PMPF cases. It is suggested that imaging methods and immunohistochemistry be combined to reduce the possibility of misdiagnosis or missed diagnosis, which may prove to be extremely beneficial in clinical practice.

## Data Availability

The original contributions presented in the study are included in the article/supplementary material. Further inquiries can be directed to the corresponding author.
